# Supplementary material for: Communities’ perceptions towards cervical cancer and its screening in Wolaita zone, southern Ethiopia: A qualitative study
Source: PLoS One. 2022 Jan 7;17(1):e0262142. doi: 10.1371/journal.pone.0262142 (PMC8740975; doi:10.1371/journal.pone.0262142)
Supplement: S1 File — (DOCX) [file pone.0262142.s001.docx]

# INFORMED CONSENT

Dear respondent my name is _____________________I am from Wolaita Sodo University. As part of the research and community service we are doing research on cervical cancer which is becoming already killer among women in Ethiopia. We are doing research entitled with community perceptions of cervical cancer screening on Southern region, Ethiopia to explore communities’ participation in cervical cancer screening. You are being asked to participate in this study because you have particular knowledge and experience that is important for this study. I assure you that all information collected from you will be confidential. The information you will provide will help achieve the objectives. Moreover, we hope to communicate findings through publication and to communicate with decision makers at regional level to implement interventions that will help to improve maternal health. The discussion shall take 1:00 to 1:30 hr. You are free to withdraw at any time in between the discussion and there will be no consequence for it.

Shall I start? Yes_________ No___________

Thank you so much

Facilitator

Date ______________time___________________ Place______________________

Socio-demographic characteristics of the respondents

| **No** | **Age** | **Sex** | **Marital status** | **Educational level** |
| --- | --- | --- | --- | --- |
|  |  |  |  |  |
|  |  |  |  |  |
|  |  |  |  |  |
|  |  |  |  |  |
|  |  |  |  |  |
|  |  |  |  |  |
|  |  |  |  |  |
|  |  |  |  |  |
|  |  |  |  |  |

**IDI**

1. Rapport building, would you tell us about one culture practice in your community that you think unique?
2. How do you explain cervical cancer?

Perceived causes of cervical cancer? Is cervical cancer curable? What is perceived cause of cervical cancer? Probe what cultural beliefs?

1. Perceived susceptibility of cervical cancer (CCA)(risk perception)

Who are at risk of getting CCA? Whom do you think are at risk of getting CCA? age

What are the risk factors for cervical cancer? What do you think might put mothers at higher risk of CCA?

1. How is it seriousness/severity? Whom do you think should be get screened for cervical cancer (married, those who had babies?

How often do you think that women should have cervical cancer screening?

1. Attitude towards CCA

What is the attitude of the community towards CCA? CCA screening?

What is the communities’ motivation towards cervical cancer? What motivates them to get screened?

What are the benefits/advantages of being screened for CCA? In what way

Where would be best place for mothers for cervical cancer screening?

1. What are facilitators for cervical cancer screening?
2. What are challenges of cervical cancer screening?
3. What are barriers to cervical cancer screening (would you mention barriers for cervical cancer screening) probe cultural barriers, accessibility of services? Time?

Who are the influential in decision making for screening?

How do husbands/partner would feel about having a pelvic examination?

Do you think it is possible to overcome those barriers? How much is it easy /or difficult to overcome those barriers?

What would make it easier for women to go for cervical cancer screening?

Where do mothers go for treatment? Probe: health facilities, traditional healers? Religious places/churches, holy water?

1. Prevention of cervical cancer

How can cervical cancer be prevented?

What needs to be done for the prevention of cervical cancer?

What should be done to facilitate or motivate mothers to go for screening/check-up?

1. Any final comment?

**FGD**

1. Rapport building, would you tell us about one culture practice in your community that you think unique?
2. How do explain cervical cancer? Probe what do you know about cervical cancer? Its causes?
3. How does it feel to be diagnosed with cervical cancer? Why?
4. How do communities define cervical cancer in your area? Can you explain that further?
5. Perceived susceptibility of cervical cancer (CCA) (risk perception) & perceived severity

Who are at risk of getting CCA? Whom do you think are at risk of getting CCA? Why?

What are the risk factors for cervical cancer? What do you think might put mothers at higher risk of CCA?

1. What are barriers to cervical cancer screening (would you mention barriers for cervical cancer screening) probe cultural barriers, accessibility of services/Facility related? Time?
2. What are the benefits/advantages of being screened for CCA? In what way? Does treatment have any benefit?
3. How do you think cervical cancer screening be delivered to your community?
4. Any final comments?
