# Supplementary material for: Communities’ perceptions towards cervical cancer and its screening in Wolaita zone, southern Ethiopia: A qualitative study
Source: PLoS One. 2022 Jan 7;17(1):e0262142. doi: 10.1371/journal.pone.0262142 (PMC8740975; doi:10.1371/journal.pone.0262142)
Supplement: Attachment — Submitted filename: REVIEW REPORT.docx [file pone.0262142.s002.docx]

**REVIEW REPORT:** **Communities’ perception of cervical cancer and screening in Wolaita zone, southern Ethiopia: A qualitative study**

The paper is a qualitative study entitled “communities ‘perception of cervical cancer and screening in Wolaita zone Southern Ethiopia “. This title is quite clear and precise. The abstract is well detailed and addresses the main research question. The study addressed one main objective namely; to explore communities’ perception of cervical cancer and screening among women in Wolaita zone, southern Ethiopia. The manuscript is an excellent demonstration of views and attitudes of a rural area population towards cervical cancer and screening. In their paper the authors used Focus Group Discussions (FGDs) and In Depth Interviews (IDI) to collect data from 59 participants living in Southern Ethiopia. Data collected was on knowledge about cervical cancer, its symptoms perception and attitude towards screening. The study was conducted in two urban and two rural *Kebeles* –which is the lowest administrative unit in Ethiopia. The sample of the study comprised of non-health professionals who had not had any training on cervical cancer residing in the study area. A proportional number of FGDs and IDIs were held in the two selected districts. Data was qualitatively presented.

The results of the study revealed that most participants had not heard about cervical cancer but had knowledge on	cancer in general. Though cervical cancer was perceived to be incurable most participants	assumed that they were not vulnerable to the disease and therefore screening was not necessary.

The authors conclude that rural communities in the zone had limited knowledge about cervical cancer and even less about risk factors, screening, treatment and prevention and therefore recommended that there is a great need for cancer education and prevention in Ethiopia.

The strength of this manuscript is that it addresses a pertinent issues concerning cervical cancer, presents findings and as such the paper represent a community’s view which will influence policy in Ethiopia and generally increase knowledge on cervical cancer screening in Sub Saharan Africa. This manuscript does an excellent job in researching on cervical cancer among non -medical/ health participants. Though an issue affecting women, the authors did an excellent job to include men in their study, this brings out a community’s holistic view on cervical cancer. Cervical cancer being a threat in sub-Saharan Africa with screening uptake being very low, this study is very relevant in highlighting some of the barriers to cervical cancer screening. The study is therefore quite relevant and is an attempt in filling the research gap as far as cervical cancer and screening are concerned. The paper however fails to discuss the interpretation and implications of the findings and secondly it does not strongly bring out the new knowledge bone from the research.

While the methodology section provides some information, it is still unclear whether the authors used the same participants for both FGDs and IDI and if so what new findings did each provide. No differences were brought out concerning the general findings between gender and age yet the authors had stratified the population based on age and gender.

Based on the findings I state that though the topic is quite relevant, it is important for the authors to address the key concerns raised so as to attract wider readability after publication. Im available to review a corrected manuscript.

**2. Discussion of specific areas for improvement**

**A. Major**

The scope of the study is well provided and the presentation is consistent throughout the manuscript. Though the manuscript is well written and nicely presented descriptively, the area of the study setting is well described and brings out the necessity for this kind of study. Though this manuscript presents a balanced assessment of cervical cancer and shows evidence of being well researched, the authors were mean with pertinent information and therefore the following areas need some revision;

- The findings that the study produces	are very critical but have no implications, and they only	need one or two sentences to bring out the implication of each findings.
- The manuscript is well written with key subtopics availed. The authors however need to be more analytical in data presentation. The excerpts are presented and not interpreted at all. Making it difficult to make exact meaning from the excerpts. It is always good practice to interpret all findings, this makes it easy to come up with the implications of the findings. The manuscript would be more useful to a broader readership if the authors moved from just providing results to interpreting them and giving the implication as well.
- I tend to think that the authors oversimplified the findings thus inhibiting the deeper message that the subject under study could provide.
- The citations should also have deeper analysis rather than simply being quoted, they should be linked to the study either as an agreement in findings or a deviation from the findings.
- The manuscript provides a recommendation but has no summary on suggested direction that people in the study area need to take and as such there is no takeaway.

**Background**

- Line 46- What is the source of this definition?
- Line 50- Any woman who is sexually active is at risk of getting HPV- this needs to be explained further so that it does not appear hanging.
- Some brief literature review of a country where cervical cancer is low could improve the background.

**Sampling size and sampling technique**

- How did you get the sample? What was the inclusion and exclusion criteria?
- Line 86-state why the two districts were selected to participate in this study ( Boloso Sore & Sodo Zuriya).
- Line 110-112 – 4 females and 2 male nurses were trained- are they research assistants? If so explain why they had all to be nurses to participate in data collection.
- Line 136- which themes were they- mention the themes here to complete this statement.
- Line 153-154 Awareness about cervical cancer- authors talk of ‘most participants’-though this is a qualitative study, it does no wrong to mention roughly how many participants instead of using the term “most”.
- Line 158- Authors talk about misconceptions but only present one misconception from the FGDs and not IDIs do the authors imply that all the respondents had similar misconceptions? Secondly, what is the implication of these misconceptions?

**Data collection procedures**

- It is not clear whether they are the same participants who were in the FGDs and interviews? Explain to clarify this. If they were different how did you choose those to participate in the FGD and who to participate in the IDI? If they participated in both what new information were you looking for and how did you carry out this? How many participants did you get per area?
- L118- authors have used the term “Unapproachable’’ I advise that they get a better term that is not judgmental.
- Line 120-121-Make it clearer- which collectors? Are they data collectors? - A bit confusing. Did the data collection exercise take place concurrently in both districts or did the same researchers finish work in one district before going to another.

**Data management**

- Line 127-129- which quality control procedures did the authors establish for their study?

**Results**

- Table 1 – needs some write up to explain the figures rather than simply putting the table and leaving it that way,
- It is important to state how many participants were in IDIs
- Did all the 59 participate in IDIs?

**Line 166- 183 Risk factors for cervical cancer**

This section is well explained though again the urban responses are not distinguished from the rural areas yet such distinctions can be quite important for recommendations

Line 181-183 talks about the men FGDs what came out from the women FGDs? This would also bring out gender differences as far as views on cervical cancer are concerned thus enriching the study. The authors had also mentioned earlier that younger men/women were in different FGDs- what was the difference in the findings based on age?

**Line 184-200-Awareness about symptoms of cervical cancer**

What are the implications of these awareness of the symptoms?

**Line 201-Perception about cervical cancer screening**

This section should bring out differences based on gender age and location (urban & rural) and also between the two districts of Wolaita zone- namely Boloso sore & Sodo Zuriya as well as the two urban and two rural *Kebeles* to strengthen the findings. If the findings are similar in these regions, what is the implication? What if they are different? Do the authors imply that all the 9 FGDs and 8 IDIs had similar findings?

**Line 229- prevention and treatment of cervical cancer**

Let the authors bring out the key points concerning cervical cancer prevention as discussed by

i) The urban dwellers
ii) Rural dwellers
iii) Young girls
iv) Young men
v) Older men/women

- What are the implications of the same to treatment? Even if it is a qualitative study it needs to be detailed in presentation of results so as to clearly bring out the implications of the findings.
- Though it is a quantitative study, the authors should avoid using terms like “several, many” etc just state how many. And where possible put figures or percentages or eg out of the 4 FGDs, 3 were of the view that e.g cervical cancer is similar to HIV/AIDS.

**Risk factors for cervical cancer**

- Line 175-180-Interpret the findings and give meaning to what the participants mean by talking about womb expulsion.
- Line 206-208- what is the implication of such results?
- Interpret the excerpts and give their implication eg line 221-228- looks disjointed when not explained before getting into another section.
- Line 245- What do the authors mean by “any other free method of screening”? Explain further, is it the financial free method or is it the screening procedure that the participants prefer.

**Discussion**

Line 262-264 what does this imply based on this study findings?

Line 265-266 what was the most common finding of these studies that was common to the authors’ study?

Line 264- link this to your study. Did the authors also establish in their study that women of low socio-economic status were at a higher risk of cervical cancer? If so according to their study what is the contributing factor and what new information came from this study?

Line 265-Let it be pelvic examination rather than pelvic exam

Line 274-275- after noting the importance what were the results or the response?

- Discussion section should be improved by linking the study findings to earlier works on the subject area.

**Minor Issues**

- The study needs quick edit, there are a few mistakes on typos, tenses and spellings
- There are a few minor observations especially on repetition
- The study did not have any funding- check financial disclosure section- which states-the authors received no specific funding for this work pg 2 then on pg 22 line 302 - Wolaita Sodo University provided funding for this study, so which is which? Or what is the authors’ interpretation of funding?
