# Supplementary material for: Communities’ perceptions towards cervical cancer and its screening in Wolaita zone, southern Ethiopia: A qualitative study
Source: PLoS One. 2022 Jan 7;17(1):e0262142. doi: 10.1371/journal.pone.0262142 (PMC8740975; doi:10.1371/journal.pone.0262142)
Supplement: Attachment — Submitted filename: Response to Reviewers.docx [file pone.0262142.s004.docx]

22/09/2021

Violet Naanyu, PhD
Academic Editor
PLOS ONE

**RE: [PONE-D-21-21479R1]: Communities’ perceptions of cervical cancer and screening in Wolaita zone, southern Ethiopia: A qualitative study**

Dear Dr Naanyu,

Once again, we would like to thank you for considering our manuscript and for arranging for it to be reviewed by reviewer(s). We have tried to address your comments and the comments / suggestions from the reviewer(s). In the ‘Response to Reviewers’, we copy each of the comments and provide the **RESPONSE** underneath. We also provide a marked-up copy of the manuscript that highlights changes made to the original version and this is uploaded as a separate file called “Manuscript with Track Changes”. Finally, we provide an unmarked version of the revised manuscript without tracked changes and this is uploaded as a separate file labelled 'Manuscript'.

We have been carefully through the peer review and have revised our paper accordingly. We feel that the paper is much improved as a result of this peer review process, and thank you for taking it to this stage.

While hoping that these changes would meet with your favourable consideration, we hold ourselves at your entire disposition for any further information or other changes you might require.

Best wishes

Gedion Asnake Azeze, on behalf of the co-authors

**Journal requirement’s:**

Please review your reference list to ensure that it is complete and correct. If you have cited papers that have been retracted, please include the rationale for doing so in the manuscript text, or remove these references and replace them with relevant current references. Any changes to the reference list should be mentioned in the rebuttal letter that accompanies your revised manuscript. If you need to cite a retracted article, indicate the article’s retracted status in the Reference list and also include a citation and full reference for the retraction notice.

**Response:**

We have reviewed our reference list and we have checked and updated all the URL links.

**Response to reviewers**

We would like to thank both the reviewer for reviewing this paper and for their comments and suggestions.

**Reviewer #1**

Right from title, the authors should refer to perceptions – It can be one perception among many communities –well within one community the perceptions are many.

**Response:**

Thank you. We have addressed this comment during the first revision and we have also checked the consistency in this current revision.

**Reviewer #2**

The authors have addressed all the concerns raised. Just polish up grammatical errors like line 138, line 172-173 and line 244.

**Response:**

We have made revisions to correct grammar and language use throughout the document. In this revised manuscript, we also believed we improved the overall tone and readability. We hope that our revisions meet your expectations.
